# Sex‐related differences in intrinsic myocardial properties influence cardiac function in middle‐aged rats during infarction‐induced left ventricular remodeling

**DOI:** 10.14814/phy2.12822

**Published:** 2016-06-10

**Authors:** Eduard I. Dedkov, Yevgen Bogatyryov, Kristina Pavliak, Adora T. Santos, Yue‐Feng Chen, Youhua Zhang, Alessandro Pingitore

**Affiliations:** ^1^ Department of Biomedical SciencesNew York Institute of Technology (NYIT) College of Osteopathic MedicineOld WestburyNew York; ^2^ National Research Council (CNR) Institute of Clinical PhysiologyPisaItaly; ^3^ Present address: Department of Internal MedicineDivision of CardiologyTexas Tech University Health Sciences CenterEl PasoTX 79905

**Keywords:** Cardiac function, left ventricular remodeling, myocardial infarction, myocardial properties, sex‐related differences

## Abstract

We previously determined that residual left ventricular (LV) myocardium of middle‐aged rats had sex‐related differences in regional tissue properties 4 weeks after a large myocardial infarction (MI). However, the impact of such differences on cardiac performance remained unclear. Therefore, our current study aimed to elucidate whether sex‐related changes in MI‐induced myocardial remodeling can influence cardiac function. A similar‐sized MI was induced in 12‐month‐old male (M‐MI) and female (F‐MI) Sprague–Dawley rats by ligation of the left coronary artery. The cardiac function was monitored for 2 months after MI and then various LV parameters were compared between sexes. We found that although two sex groups had a similar pattern of MI‐induced decline in LV function, F‐MI rats had greater cardiac performance compared to M‐MI rats, considering the higher values of EF (39.9 ± 3.4% vs. 26.7 ± 7.7%, *P* < 0.05), SW index (40.4 ± 2.1 mmHg • mL/kg vs. 20.2 ± 3.3 mmHg • mL/kg, *P* < 0.001), and CI (139.2 ± 7.9 mL/min/kg vs. 74.9 ± 14.7 mL/min/kg, *P* < 0.01). The poorer pumping capacity in M‐MI hearts was associated with markedly reduced LV compliance and prolonged relaxation. On the tissue level, F‐MI rats revealed a higher, than in M‐MI rats, density of cardiac myocytes in the LV free wall (2383.8 ± 242.6 cells/mm^2^ vs. 1785.7 ± 55.9 cells/mm^2^, *P* < 0.05). The latter finding correlated with a lower density of apoptotic cardiac myocytes in residual LV myocardium of F‐MI rats (0.18 ± 0.08 cells/mm^2^ vs. 0.91 ± 0.30 cells/mm^2^ in males, *P* < 0.01). Thus, our data suggested that F‐MI rats had markedly attenuated decline in cardiac performance compared to males due to ability of female rats to better retain functionally favorable intrinsic myocardial properties.

## Introduction

Myocardial infarction (MI) remains one of the major causes of systolic heart failure (HF) in the elderly of both sexes (Hellermann et al. 2002; Weir et al. 2006). In patients with an acute MI, the impairment of left ventricular (LV) function, which is initially caused by severe ischemic myocardial damage, has often progressed to HF due to adverse LV chamber remodeling (Gaasch et al. 2008). During this process, the alteration in LV geometry and mass (Gaasch and Zile 2011) has been often accompanied by significant changes in myocardial tissue properties (Beltrami et al. 1994).

In recent years, a number of clinical trials have reported that among patients with systolic HF, women have lower risk of cardiovascular mortality than men (Frazier et al. 2007; O'Meara et al. 2007; Dunlay and Roger 2012). To some extent, this phenomenon has been associated with sex‐related differences in myocardial tissue alterations during post‐MI ventricular remodeling (Piro et al. 2010), particularly, with the lower rate of cardiac myocyte death (Biondi‐Zoccai et al. 2005) and smaller magnitude of cardiac myocyte hypertrophy (Crabbe et al. 2003) within residual myocardium of the female patients. Despite that, there are data from other clinical studies which contradict these findings, suggesting that women with HF or severe LV dysfunction of ischemic etiology had either similar or even worse survival compared to men (Vaccarino et al. 2001; Kwon et al. 2009; Lam et al. 2015). In large part, such inconsistencies might arise from the fact that majority of gender‐related clinical trials have enrolled patients based primarily on the severity of systolic LV dysfunction without taking into consideration any potential changes in myocardial tissue properties (O'Meara et al. 2007).

Similarly, small animal models of experimental systolic HF has also revealed the contradictory results, with some studies partially supporting (Litwin et al. 1999; Jain et al. 2002; Wu et al. 2003; Cavasin et al. 2004; Wang et al. 2007; Shioura et al. 2008; Dedkov et al. 2014) and others mostly opposing (Bridgman et al. 2005; Chen et al. 2011; Sofia et al. 2014; Antonio et al. 2015) the influence of biological sex on the MI‐induced LV remodeling process. Moreover, those studies in which the residual LV myocardium was further evaluated on the tissue level have also shown a high degree of discrepancy regarding the sex‐related changes in principal properties of the myocardial tissue, such as the size of cardiac myocyte and the content of interstitial collagen (Litwin et al. 1999; Cavasin et al. 2004; Bridgman et al. 2005; Chen et al. 2011; Dedkov et al. 2014). For the most part, the inconsistencies among results from these studies could be attributed to the differences in key experimental variables, such as the species, strains, and age of animal used as well as the initial MI size and the duration of the remodeling process. On the other hand, the lack of uniform methodological approaches among previously published studies, especially, in evaluating structural LV parameters could also obscure a proper depiction of sex‐related changes which occurred in surviving myocardium after MI.

In our recent study on middle‐aged rats, we found that although both sexes had undergone a relatively analogous pattern of global LV remodeling during 4 weeks following a large similar‐sized MI, the residual LV myocardium from male and female rats had obvious regional differences in the scales of cardiac myocyte hypertrophy, interstitial fibrosis, and adaptive arteriogenesis (Dedkov et al. 2014). Unfortunately, this earlier study was not aimed to evaluate the impact of sex‐related differences in structural LV alterations on the level of post‐MI cardiac dysfunction and, therefore, this matter remained unclear. Furthermore, the interpretation of our findings has been complicated by the fact that the rest of the relevant experimental data from other research groups were obtained on either young or young‐adult animals (Litwin et al. 1999; Cavasin et al. 2004; Bridgman et al. 2005; Wang et al. 2007; Shioura et al. 2008; Chen et al. 2011; Sofia et al. 2014; Antonio et al. 2015). This is surprising, considering that there are compelling evidences demonstrating that normal aging in rodents, such as rats, can markedly affect the myocardial tissue properties (Forman et al. 1997; Raya et al. 1997; Nitahara et al. 1998) as well as the functional capacity of the heart (Boluyt et al. 2004). Moreover, taking into consideration that the risk of MI is markedly higher in middle‐aged and senescent individuals, the evaluation of sex‐related changes in post‐MI hearts of middle‐aged, rather than young, animals is of greater relevance to humans.

Accordingly, we designed our study in order to repeatedly assess the pattern of changes in LV chamber geometry and systolic function between male and female middle‐aged rats at several time points during 8 weeks following a large, similar‐sized MI. Furthermore, we intended to determine whether global cardiac changes would correlate with distinctive sex‐related differences in intrinsic tissue properties of the remaining, noninfarcted LV myocardium.

## Materials and Methods

All animal procedures were performed in compliance with the *Guide for the Care and Use of Laboratory Animals* (National Institute of Health Publications No. 85‐23, revised 1996) and were approved by the Institutional Animal Care and Use Committee at New York Institute of Technology College of Osteopathic Medicine.

### Animals and experimental protocol

The experiments were conducted on 12‐month‐old (middle‐aged) male (*n* = 10) and female (*n* = 10) Sprague–Dawley rats (Charles River Laboratories, Inc., Wilmington, MA). At the beginning of the experiments, all rats were weighed and evaluated by echocardiography to assess the LV dimensions, chamber volumes, myocardial mass, wall thickness, and systolic function at baseline. Then, a large transmural myocardial infarction (MI) was induced under ketamine (100 mg/kg intraperitoneal [*i.p*.]) and xylazine (10 mg/kg *i.p*.) anesthesia by the permanent ligation of the left anterior descending branch of the coronary artery, as previously detailed (Dedkov et al. 2005). Following surgery, the rats were housed under climate‐controlled conditions at a 12‐h light/dark cycle, and provided with standard rat chow and water ad libitum. The mortality rate among post‐MI rats was ~30% in males and ~10% in females, with all death occurring within first 48 h after surgery.

Two weeks after induction of an MI, an echocardiographic examination was repeated in all survived rats to measure the infarcted area. The animals were included in the following study only if the size of transmural MI was greater than ~35% of the LV circumference (Pfeffer et al. 1979). In all selected post‐MI male (M‐MI) and female (F‐MI) rats, the body weight and the same LV echocardiographic parameters were re‐examined with a 2‐week interval between second and eighth post‐MI week.

At the end of experiment, each rat was acutely instrumented with retrograde catheters in the left ventricle and the right atrium to examine systemic and cardiac hemodynamics, LV systolic and diastolic properties, and to perform an atrial fibrillation (AF) inducibility test, respectively (Zhang et al. 2013, 2014). Finally, animals were killed by arresting the heart in diastole with potassium chloride; the body was weighed, and the hearts and tibias were collected for further evaluation.

### Two‐dimensional echocardiographic examination

Two‐dimensional (2‐D) parasternal short‐ and long‐axis views of the left ventricle were obtained using a GE Vivid 7 Dimension Ultrasound System (GE Vingmed Ultrasound, Horten, Norway) coupled with a M12L linear (Matrix) array ultrasound transducer probe (5.5–14 MHz), as previously described (Zhang et al. 2013, 2014). Briefly, the rat was anesthetized with isoflurane in pure oxygen (3% for induction and 1.5% for maintenance) and weighed, its anterior and left lateral thoracic regions were shaved and the rat was placed on an isothermal pad maintained at ~40°C in the left lateral decubital position. The size of the infarcted area was estimated from the 2‐D short‐axis view at the level of the papillary muscles as the percentage ratio of the length of the akinetic (infarcted) area to the LV circumference. From the same short‐axis view, 2‐D guided M‐mode tracings were recorded to measure anterior and posterior wall thickness in end‐diastole and end‐systole as well as LV end‐diastolic and end‐systolic internal dimensions. For each M‐mode tracings, at least three consecutive cardiac cycles were sampled. The LV mass, end‐diastolic, and end‐systolic volumes were calculated by the area‐length method. All measurements were done offline using the GE EchoPAC SW BT11 software (GE Vingmed Ultrasound, Horten, Norway). Pulse‐wave Doppler interrogation of mitral inflow was used to determine the heart rate. From acquired measurements, relative wall thickness, LV mass‐to‐end‐diastolic volume ratio, stroke volume, stroke work, cardiac output, posterior wall thickening, fractional shortening, and ejection fraction were calculated with standard equations (Lang et al. 2015). Left ventricular end‐systolic and end‐diastolic meridional wall stress were calculated from M‐mode measurements in combination with LV pressure data using the previously validated formula (Reichek et al. 1982): (0.334 × LV pressure × LV Internal Diameter)/(Posterior Wall Thickness × [1 +  Posterior Wall Thickness/LV Internal Diameter]). Considering the fact that body weight and heart size were greater in male than in age‐matched female rats, LV internal dimensions, mass, end‐diastolic and end‐systolic volumes, stroke volume, and cardiac output were normalized to body weight in order to assess these parameters independently of the effect of different body size between two sex groups.

### Systemic and left ventricular hemodynamic measurements

Systemic and LV hemodynamics were obtained by retrograde catheterization of the right carotid artery with the use of a 1.9 F rat pressure‐volume catheter (Scisense, London, Ontario, Canada), as previously described (Zhang et al. 2013, 2014). Briefly, the rat was anesthetized with isoflurane in pure oxygen (3% for induction and 1.5% for maintenance) and the catheter was inserted into the right carotid artery. After systolic, diastolic, and mean arterial pressure were measured, the tip of the catheter was advanced through the aorta into the LV cavity and the following cardiac parameters were acquired: heart rate, stroke work, end‐systolic pressure, peak systolic pressure, end‐diastolic pressure, maximum positive and negative change in pressure over time (+d*P*/d*t* and −d*P*/d*t*), and the time constant for isovolumic relaxation (Tau). The data were acquired using an ADVantage PV system (Transonic Systems Inc., Ithaca, NY) and analyzed with a LabScribe 3 software (iWorx Systems, Dover, New Hampshire).

### Atrial fibrillation inducibility test

An atrial fibrillation (AF) inducibility test was performed with the use of a 1.6 F octopolar electrophysiology catheter (EPR‐802, Millar Instruments, Inc., Houston, Texas) as previously described (Zhang et al. 2013, 2014). Briefly, the rat was anesthetized with isoflurane in pure oxygen (3% for induction and 1.5% for maintenance) and the catheter was inserted into the right jugular vein and advanced into the right atrium. The electrocardiograms from a standard surface lead II and three pairs of right atrial electrodes were acquired using PowerLab data acquisition systems and LabChart 7 Pro software (ADInstruments, Colorado Springs, Colorado). While the electrocardiograms from distal, middle, and proximal pairs of electrodes were used to facilitate determination of atrial capturing and AF pattern, the electrode poles 5 and 6 were used for atrial pacing.

AF was induced by atrial burst pacing with 200 impulses at 50 Hz. The duration of the subsequent spontaneous arrhythmias after burst pacing was recorded. For each rat, the average arrhythmia duration was calculated based on five such tests. AF was defined as irregular rapid atrial activations, typically >1500 beats/min, with varying electrographic morphology lasting ≥0.5 sec, as previously described (Zhang et al. 2013, 2014).

### Tibia length and ventricular weight measurement, infarct size estimation, and tissue collection

The tibia was dissected from one of the hindlimbs, as previously reported (Yin et al. 1982), and its length, from the condyles to the tip of the medial malleolus, was measured with a digital caliper.

The heart was excised and perfuse‐fixed on a Langendorff apparatus under constant pressure (100 mmHg) for 30 min with a precooled solution of 4% paraformaldehyde (PFA) in phosphate‐buffered saline (PBS). Then, the heart was immersed in a fresh solution of 4% PFA in PBS and stored for 24 h at 4°C. At the end of this period, the atria and great vessels were trimmed off and the ventricles were transferred to PBS. In each heart, the right ventricular free wall was carefully cut off from the left ventricle (LV free wall plus septum) with scissors and then the left ventricle was transversely cut into five parallel slices with a multiblade guillotine. The pieces of RVFW and LV slices were briefly blotted dry with filter paper and separately weighed.

In each left ventricle, all transverse slices were digitized using a Motic K‐400L stereo microscope (Motic Instruments, Inc., Richmond, BC, Canada) equipped with an Olympus DP70 digital camera (Olympus America, Inc., Center Valley, MA) and infarct size was estimated using Image‐Pro Analyzer 7 software (Media Cybernetics, L.P., Silver Spring, MD), as detailed previously (Dedkov et al. 2014). Briefly, in each digitized LV slice, the lengths of the circumference, the free wall, and a portion occupied by the scar (all obtained at the mid‐wall level) were measured. The extent of the scarred area was estimated in each slice, first, as the percentage ratio of the length of the scar to the LV circumference and, second, as the percentage ratio of the length of the scar to the length of the free wall. Finally, the infarct size in the entire left ventricle was expressed as the mean percentage of the LV circumference and the LV free wall, respectively.

From each heart, two midventricular slices (at the level of the papillary muscles) were processed and embedded in paraffin, as previously reported (Dedkov et al. 2014).

### Histology, immunohistochemistry, TUNEL assay, and microscopy

Transverse 8.0‐*μ*m‐thick serial sections were cut from paraffin‐embedded LV slices onto microscope slides. One group of sections were processed with the standard histological methods, including hematoxylin and eosin (H&E), Masson's trichrome, toluidine blue, and picrosirius red stains. A second group of sections was immunostained with a rabbit anti‐laminin antibody (1:60; cat. L9393; Sigma, St. Louis, MO) in combination with an Alexa Fluor 594‐conjugated *Griffonia* S*implicifolia* isolectin IB4 (GS‐IB4) labeling (10 *μ*g/mL; cat. I21413; Molecular Probes, Inc., Eugene, OR). An Alexa Fluor 488‐conjugated goat anti‐rabbit antibody (2 *μ*g/mL; cat. A‐11008; Molecular Probes, Inc., Eugene, OR) was used to detect a primary anti‐laminin antibody. All sections were coverslipped with ProLong Gold antifade mounting medium containing DAPI (4′, 6‐Diamidino‐2‐Phenylindole; cat. P‐36931; Molecular Probes, Inc., Eugene, OR) to visualize cell nuclei.

A third group of sections was immunostained with a mouse anti‐cardiac myosin heavy‐chain beta (MHC‐*β*) isoform antibody (1:1000; M8421; Sigma, St. Louis, MO) in combination with the TUNEL (terminal deoxynucleotidyl transferase dUTP nick‐end labeling) assay. A Rhodamine Red‐X‐conjugated goat anti‐mouse antibody (1:200; cat. 115‐295‐146; Jackson ImmunoResearch Laboratories, Inc., West Grove, PA) was used to detect a primary anti‐cardiac MHC‐*β* isoform antibody. The TUNEL assay was done with an ApopTag Plus fluorescein in situ apoptosis detection kit (cat. S7111; Chemicon Int, Temecula, CA), which uses a recombinant terminal deoxynucleotidyl transferase (TdT) to catalyze the addition of digoxigenin‐labeled dUTP nucleotides to the free 3′‐OH ends of the DNA strand breaks within nuclei of cells undergoing apoptosis. The apoptotic nuclei and apoptotic bodies were visualized by a fluorescein‐conjugated sheep anti‐digoxigenin antibody and were confirmed by nuclear counterstaining with DAPI. For a negative control, some sections were incubated in the absence of TdT enzyme, whereas for a positive control, the sections were predigested with DNase I (1 *μ*g/mL; cat. D7291; Sigma, St. Louis, MO).

The stained sections were examined under an Olympus BX53 fluorescent microscope (Olympus America, Inc., Center Valley, MA); light and fluorescence images were captured into a computer using an Olympus DP72 digital camera (Olympus America, Inc., Center Valley, MA). The composite figures were digitally assembled and annotated using Adobe Photoshop CS6 software version 13.0.1 (Adobe Systems; San Jose, CA).

### Morphometry and quantitative image analysis

Quantitative morphometric examination was conducted on digitized images using Image‐Pro Analyzer 7 software (Media Cybernetics, Inc., Bethesda, MD). All data from the remaining noninfarcted myocardium of the LV free wall were derived from the tissue ~1.5–2 mm distal from the scar edge.

The H&E and Masson's trichrome stained sections were evaluated under the medium‐power magnification (ob. ×12) using a Motic K‐400L stereo microscope (Motic Instruments, Inc., Richmond, BC, Canada) equipped with an Olympus DP70 digital camera (Olympus America, Inc., Center Valley, MA) to obtain the following parameters of the left ventricle: LV cross‐sectional area (CSA) and the mean diameter, LV cavity CSA and the mean cavity diameter, the average thickness of the free wall and interventricular septum, the average thickness and CSA of the scar. Using these measurements, the following global LV indices were computed: (1) LV cavity diameter to septum thickness ratio; (2) the scar thinning ratio (the ratio between average thickness of the scar and the average thickness of the septum); and (3) the infarct expansion index ([LV cavity area/LV area] × [septal wall thickness/scar thickness]), as previously reported (Bogatyryov et al. 2013; Dedkov et al. 2015).

Picrosirius red stained sections were used to determine the myocardial content of fibrillar collagen. Briefly, the five to six randomly selected optical fields within the myocardium of the LV free wall and septum, which did not have the profiles of arteries and veins, were digitized under the high‐power magnification (ob. ×40). The interstitial collagen content was estimated as the volume fraction of the area occupied by both fibrillar collagen and cardiac myocytes.

Toluidine blue stained sections were used to estimate the myocardial density of mast cells. Briefly, the entire profiles of the remaining myocardium in the LV free wall and septum were captured under the medium‐power magnification (ob. ×12) and their area was separately measured. Then, the total number of mast cells within each myocardial region was counted under the high‐power magnification (ob. ×40). Finally, mast cell numerical density was expressed as the cell number per area of the myocardium.

The sections co‐stained with an anti‐laminin antibody and a GS‐IB4 lectin was used to estimate the mean cardiac myocyte diameter and CSA, capillary and cardiac myocyte numerical density as well as the capillary‐to‐myocyte ratio, as previously reported (Dedkov et al. 2014). All parameters were determined separately in two myocardial regions: the LV free wall epimyocardium and the septal endomyocardium. Only the areas in which the capillaries had the transverse profiles while the cardiac myocytes showed the round‐shaped, centrally located nuclei were used for evaluation. Briefly, all the laminin‐stained profiles of the cardiac myocytes within the area were outlined to determine the mean myocyte diameter, the mean CSA, and numerical density, whereas GS‐IB4‐labeled capillary profiles, detected in the same areas, were counted to estimate capillary numerical density and the capillary‐to‐myocyte ratio.

The sections co‐labeled with an anti‐cardiac MHC‐*β* isoform antibody, TUNEL assay, and DAPI were used to assess the myocardial density of apoptotic (TUNEL‐positive) cells among cardiac myocytes (CMs) and noncardiac myocyte (non‐CM) cells. Briefly, the triple‐labeled fluorescence images from five to six randomly selected optical fields in the remaining myocardium of the LV free wall and septum were captured under the medium‐power magnification (ob. ×20) and digitally merged using Olympus cellSens Standard 1.13 software (Olympus America, Inc., Center Valley, MA). A cell was counted as an apoptotic CM only if the TUNEL‐positive nucleus or apoptotic bodies were superimposed on a cardiac MHC‐*β* isoform‐stained profile. All other cells, which demonstrated the TUNEL‐positive nuclei or the presence of apoptotic bodies but located in the interstitial space between cardiac MHC‐*β* isoform‐stained myocytes, were counted as non‐CM cells. The TUNEL‐positive reaction was always confirmed by the morphological appearance of apoptotic changes in DNA‐containing structures (nucleus or apoptotic bodies) using DNA‐binding dye DAPI. The total number of TUNEL‐positive CMs and non‐CM cells within each myocardial region of the heart was counted and their numerical densities were expressed as the cell number per area of the myocardium, as reported previously (Park et al. 2009). On average, approximately 2.1–2.5 mm^2^ of the myocardium per LV region (free wall and septum) were examined in each heart.

### Statistical analysis

Data are expressed as the mean ± SEM. Statistical analysis was performed using Prism 6 software package (IBM Corp., Armonk, NY). A one‐ and two‐way analysis of variance (ANOVA) followed by the Tukey's and Dunnett's post hoc tests were performed for multigroup comparisons. A two‐tailed, unpaired Student's *t*‐test was used to determine the difference between two groups. *P *<* *0.05, *P *<* *0.01, and *P *<* *0.001 were selected to denote the different levels of statistical significance.

## Results

### Echocardiographic assessment of dynamic changes in LV geometry and function

Serial examinations of LV structural and functional parameters were performed in two groups of middle‐aged male and female rats between baseline (time point 0) and eighth week following a large transmural MI. The main emphasis was made on a comparison of sex‐specific changes occurred in LV geometry and systolic performance during a 2‐month‐period of post‐MI cardiac remodeling. Because the body weight in middle‐aged female rats was significantly smaller than in age‐matched male rats for the duration of the study, by ~30% (*P* < 0.01) on average, LV internal dimensions, LV mass, end‐diastolic and end‐systolic volumes, stroke volume, and cardiac output were additionally normalized to body weight in order to compare these parameters independently of the effect of different body size between sexes. However, it is important to emphasize that in each sex group, the average body weight remained almost constant during an entire experimental period (data not shown).

**Figure 1:**
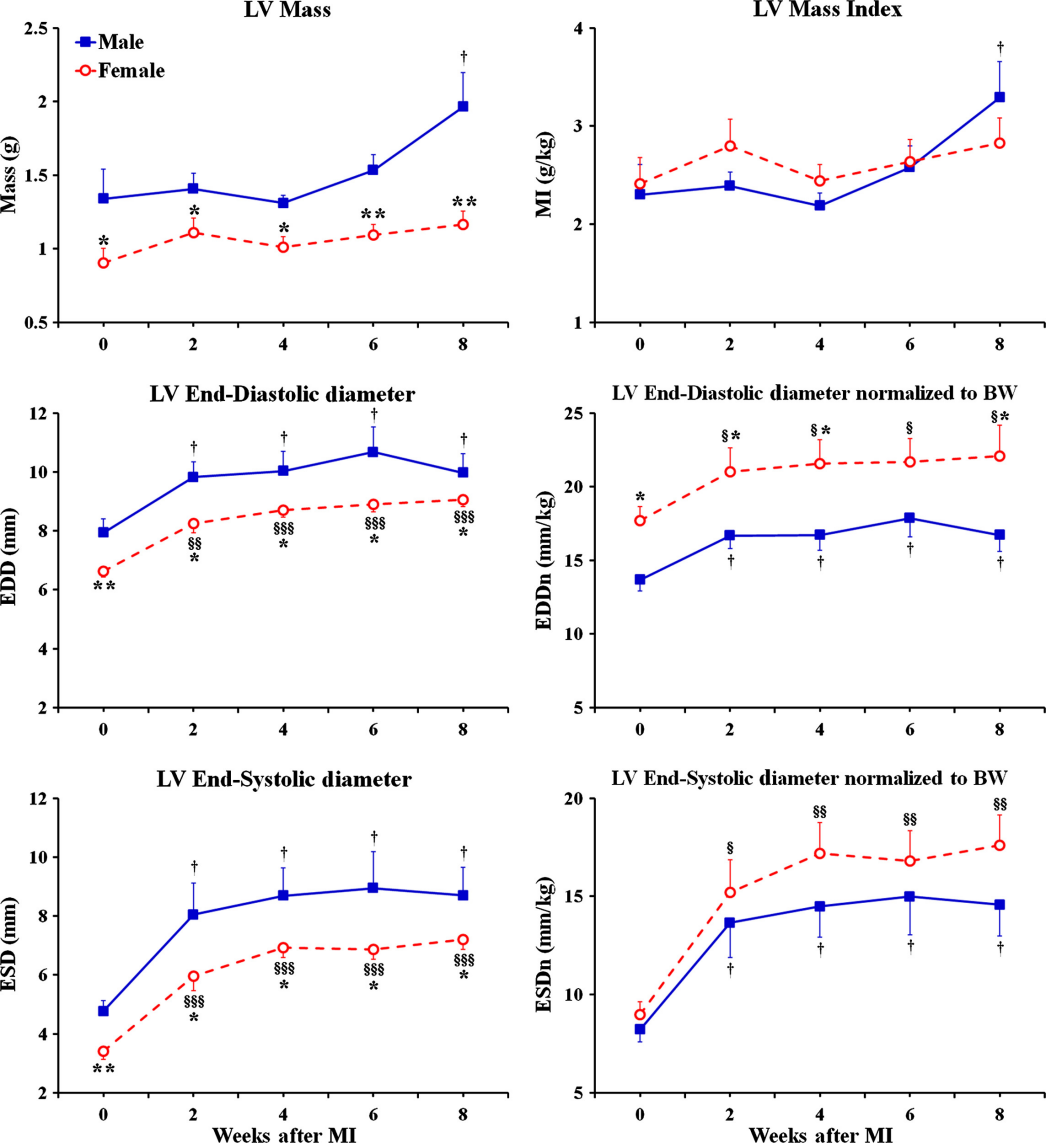
Time course of changes in LV mass and internal dimensions obtained by echocardiography in male and female middle‐aged rats during 8 weeks after MI. MI, mass index; EDD, end‐diastolic diameter; ESD, end‐systolic diameter; BW, body weight; EDDn, LV internal end‐diastolic dimension normalized to BW; ESDn, LV internal end‐systolic dimension normalized to BW. Considering that LV mass and mass index in post‐MI rats of two sex groups remained either similar or above the corresponding baseline levels during the entire study, the growth response in the remaining LV myocardium was adequate in both sexes to compensate for the lost tissue in the infarcted free wall. Data are means ± SEM;* n* = 6 male rats/group; *n* = 7 female rats/group. *P < 0.05 and **P < 0.01 females versus males at the same time point; ^§^ *P* < 0.05, ^§§^ *P* < 0.01 and ^§§§^ *P* < 0.001 F‐MI rats versus female baseline; ^†^ *P* < 0.05 M‐MI rats versus male baseline.

**Figure 2:**
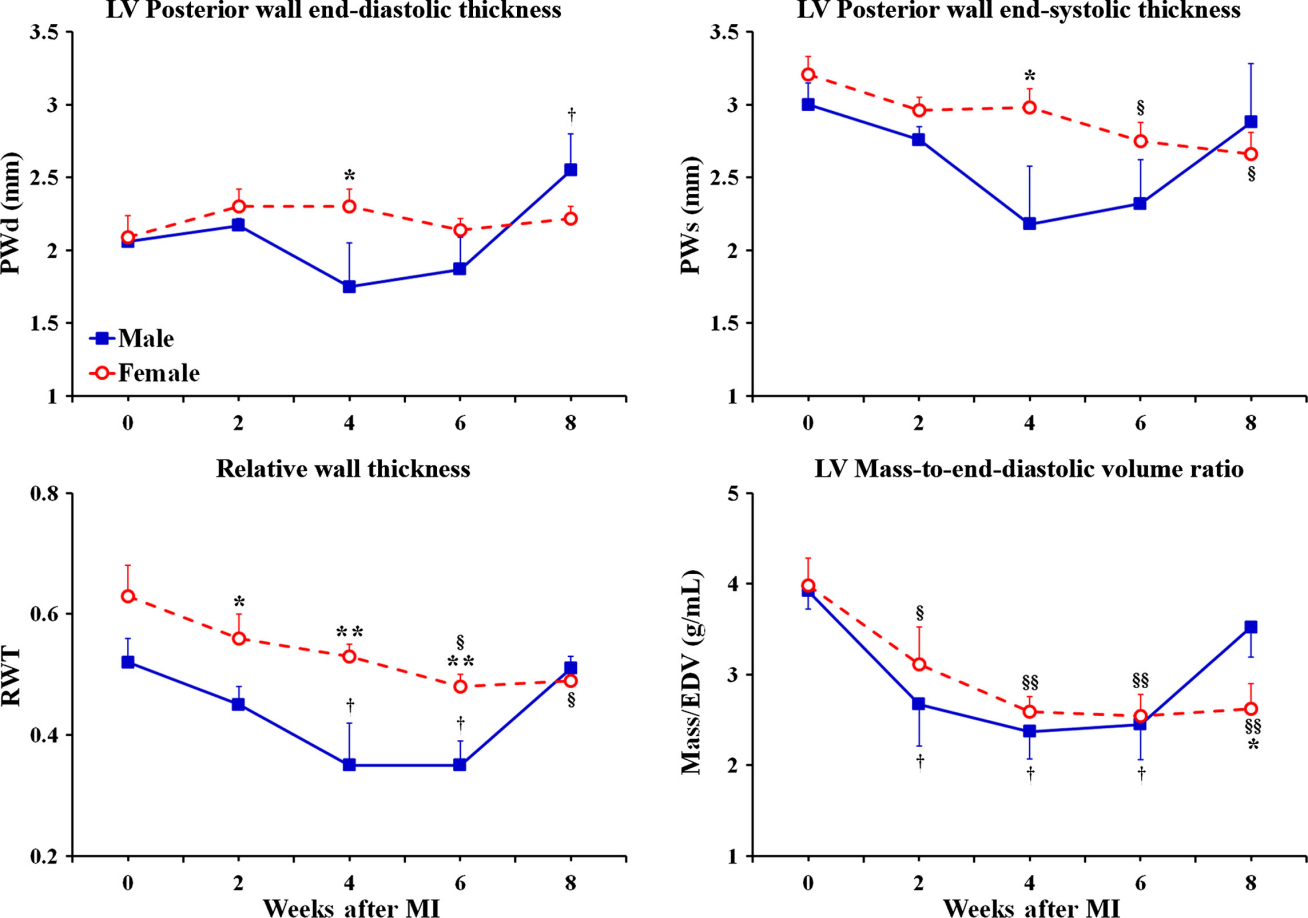
Time course of changes in the pattern of LV chamber remodeling and LV geometry obtained by echocardiography in male and female middle‐aged rats during 8 weeks after MI. PWd, posterior wall thickness at end diastole; PWs, posterior wall thickness at end systole; RWT, relative wall thickness = (2 × PWd)/EDD; EDV, LV end‐diastolic volume. The analogous reduction in RWT and LV mass/EDV ratio seen in both sex groups during 6 post‐MI weeks is characteristic to eccentric remodeling, during which the insufficient myocardial growth in the noninfarcted LV wall mismatches a marked dilatation of LV cavity. However, the return of RWT and LV mass/EDV ratio back to the baseline values detected in male rats at the end of eighth post‐MI week indicates the sudden transition of LV geometry in males, as opposed to female rats, from eccentric remodeling to eccentric hypertrophy, in which the compensatory growth of the remaining LV myocardium matches the extent of LV cavity dilatation. Data are means ± SEM;* n* = 6 male rats/group; *n* = 7 female rats/group. *P < 0.05 and **P < 0.01 females versus males at the same time point; ^§^ *P* < 0.05 and ^§§^ *P* < 0.01 F‐MI rats versus female baseline; ^†^ *P* < 0.05 M‐MI rats versus male baseline.

**Figure 3:**
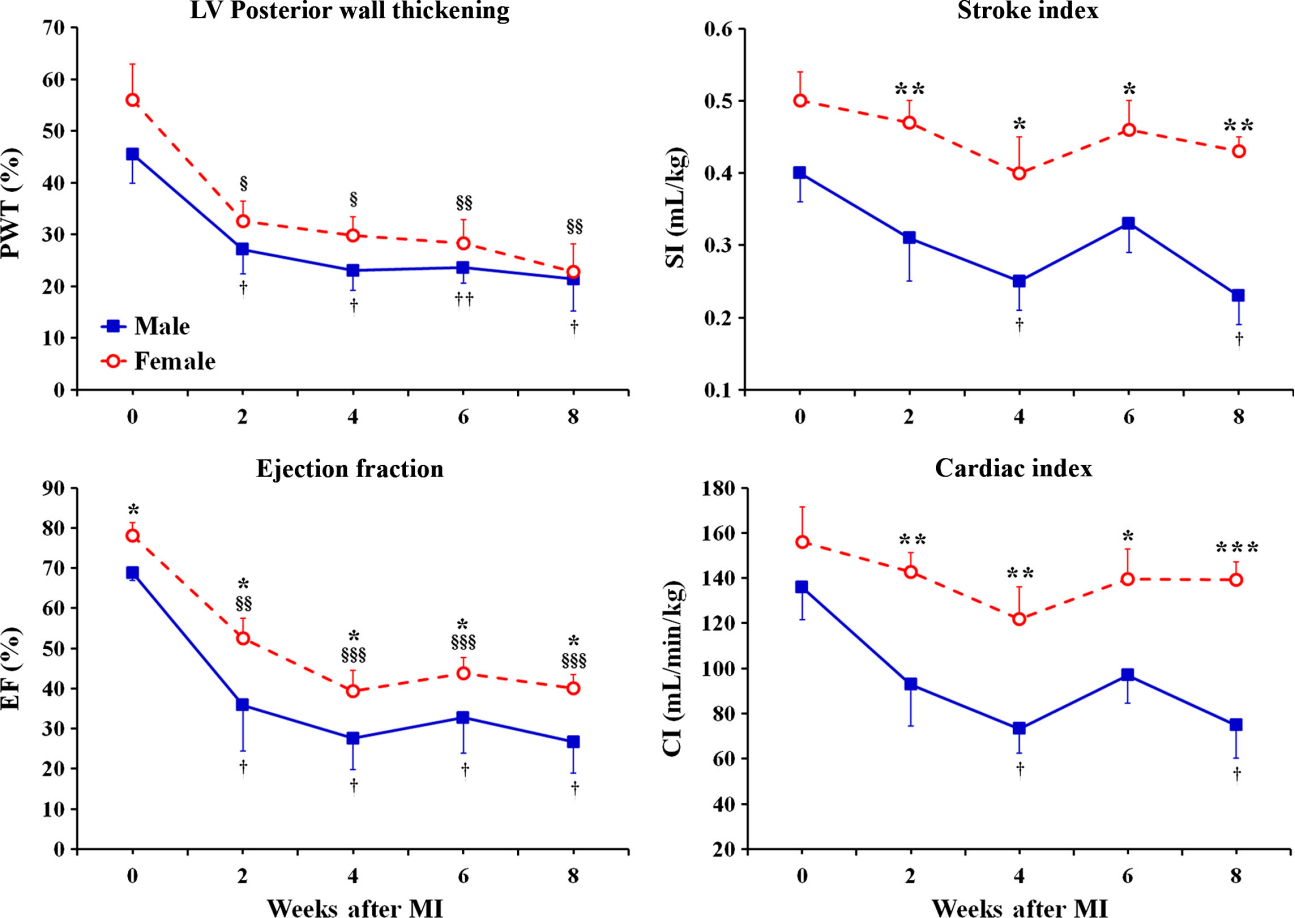
Time course of changes in regional and global LV systolic function and cardiac performance obtained by echocardiography in male and female middle‐aged rats during 8 weeks after MI. PWT, posterior wall thickening = (PWs‐PWd)/PWd; EF, ejection fraction; SI, stroke index = SV/BW; CI, cardia index = CO/BW. Although both groups of rats demonstrated the analogous decline in regional systolic function, represented by changes in PWT, the underlying mechanisms were sex specific. For instance, in male rats, the principal cause of regional systolic dysfunction was a marked increase in PWd, while, in female rats, it resulted from a progressive reduction in PWs. Note that sex‐specific differences in cardiac index occurred independently of heart rate which was similar between two sex group at each time point in the study (data not shown). Data are means ± SEM;* n* = 6 male rats/group; *n* = 7 female rats/group. *P < 0.05, **P < 0.01 and ***P < 0.001 female versus male groups at the same time point; ^§^ *P* < 0.05, ^§§^ *P* < 0.01 and ^§§§^ *P* < 0.001 F‐MI rats versus female baseline; ^†^ *P* < 0.05 and ^††^ *P* < 0.01 M‐MI rats versus male baseline.

Before the onset of MI (at baseline), the female rats demonstrated significantly smaller, than in male rats, LV mass as well as LV end‐diastolic (EDD) and end‐systolic (ESD) dimensions (Fig. 1). At the same time, the rats of both sexes had relatively comparable end‐diastolic (PWd) as well as end‐systolic (PWs) thickness of the LV posterior wall (Fig. 2). As a result, the left ventricle in female rats showed ~18% greater, than in males, relative wall thickness (RWT) at the end of diastole, suggesting lower regional end‐diastolic wall tension in female LV chamber (Fig. 2). Interestingly, when LV mass and the ESD were normalized to corresponding body weights, these parameters became almost identical between sexes, as revealed by the analogous levels for both LV mass index and the normalized ESD (Fig. 1). In contrast, the normalized EDD became ~23% (*P* < 0.05) larger in females, compared to male rats, suggesting that the LV myocardium in female rats might have better, than in males, compliance during diastole. Most important, the female rats revealed a significantly greater difference in percent change between EDD and ESD during ventricular contraction in comparison with male rats (~Δ56% vs. ~Δ40% P < 0.05, respectively). Moreover, although the rats of both sexes had similar LV chamber geometry, as was determined on the basis of the identical LV mass‐to‐end‐diastolic volume (mass/EDV) ratio (Fig. 2), in female rats, the ejection fraction (EF) and fractional shortening (FS) were significantly higher by ~12% and ~18% (*P* < 0.05), respectively, compared to male rats (Fig. 3). In addition, the levels of regional LV systolic function, that is, posterior wall thickening (PWT) as well as of the cardiac performance indices, such as stroke index and cardiac index, were also greater in females, compared to male rats (Fig. 3). All together, these findings indicated that middle‐aged female rats had better LV pumping capacity compared to age‐matched males.

During 8 weeks following a large transmural MI, the LV chamber, in rats of both sexes, has undergone the analogous structural changes, consisting primarily of marked cavity dilatation and the compensatory growth of the remaining myocardium (Fig. 1). By the end of second week after the onset of MI, the rats in both groups demonstrated almost identical expansion of LV cavity dimensions with regard to the corresponding baseline levels, as demonstrated by the uniformed, sex‐independent increase in EDD and ESD by ~24% and ~66% (*P* < 0.05), respectively (Fig. 1). At the same time, in both sexes, the LV mass remained comparable to the preinfarcted levels, suggesting that both female and male rats were able to adequately compensate for a substantial loss of cardiac myocytes by means of reactive myocardial hypertrophy in noninfarcted parts of the left ventricle. Interestingly, the compensatory myocardial growth during this period occurred in the absence of a noticeable increase in LV posterior wall thickness (Fig. 2), suggesting preferentially radial wall expansion. As a result, the rats of both sexes demonstrated a parallel decline in RWT that was coincided with a significant reduction in mass/EDV ratio, consistent with eccentric LV chamber remodeling (Fig. 2). Most important, the similar scale of changes in LV chamber geometry between sexes was associated with the relatively comparable pattern of reduction in regional and global LV systolic function with regard to the corresponding baseline levels in male and female groups of rats (Fig. 3). Nevertheless, global LV systolic function as well as cardiac performance remained markedly higher in post‐MI females, compared to male rats, as revealed by the values of EF and cardiac index, which were greater in female rats by ~32% (*P* < 0.05) and ~35% (*P* < 0.01), respectively (Fig. 3).

In the course of the following month, between weeks 2 and 6 after MI, the rats of both sexes demonstrated a relatively comparable trend in LV chamber alterations that resulted in further decrease in both the RWT and the mass/EDV ratio, suggesting continuing eccentric LV chamber remodeling in two post‐MI groups (Figs. 1 and 2). Furthermore, the rats of both sexes showed a further decline in regional and global LV systolic function with regard to the corresponding baselines (Fig. 3). However, the female rats has continued to demonstrate the significantly higher levels for EF and the indices of cardiac performance, which remained elevated above the levels in male rats by ~30% and ~35% (*P* < 0.05) on average, respectively (Fig. 3).

During the following 2 weeks, particularly, by the end of eighth week after MI, the LV chamber in male rats, in contrast to females, revealed a significant increase in LV mass and mass index, which both became ~48% (*P* < 0.05) larger of the corresponding baseline levels (Fig. 1). Such exaggerated myocardial growth in males occurred without an additional radial expansion of LV cavity and was associated with a ~24% (*P* < 0.05) increase in PWd thickness with regard to the baseline level (Fig. 2). Therefore, by the end of eighth post‐MI week, the values of both the RWT and the mass/EDV ratio, in male rats, became nearly analogous to those at the baseline, indicating that the male LV chamber has acquired the geometry consistent with eccentric hypertrophy (Fig. 2). On the other hand, in female rats, the thickness of PWd remained relatively unchanged for the duration of the study and, therefore, the female LV chamber continued to demonstrate the structural indices of LV geometry similar to the pattern of eccentric remodeling. Interestingly, despite the concentric myocardial growth in LV chamber wall, the post‐MI males continued to show a significantly smaller difference in percent change between EDD and ESD during ventricular contraction compared to post‐MI female rats (~Δ13% vs. ~Δ21% *P* < 0.05, respectively), suggesting greater ventricular stiffness in the former (Fig. 1). As a result, the male rats revealed markedly reduced EF and cardiac index, which were significantly lower than in post‐MI females by ~33% (*P* < 0.05) and ~46% (*P* < 0.001), respectively (Fig. 3). Most important, a greater cardiac index in female rats was associated with a significantly higher stroke index, indicating a better preserved stroke volume in females compared to male rats (Fig. 3).

Thus, although the progressive myocardial growth in male rats has facilitated the transition of LV geometry toward eccentric hypertrophy by the end of eighth post‐MI week, the lack of a noticeable improvement in male systolic function, in comparison with female rats, raised a possibility that the intrinsic properties of the remaining LV myocardium might carry some distinctive sex‐related differences between post‐MI male and female rats. In order to elucidate this issue, various cardiac parameters were compared between rats of two sexes at the end of the study.

### Hemodynamic assessment of LV systolic and diastolic properties

**Figure 4:**
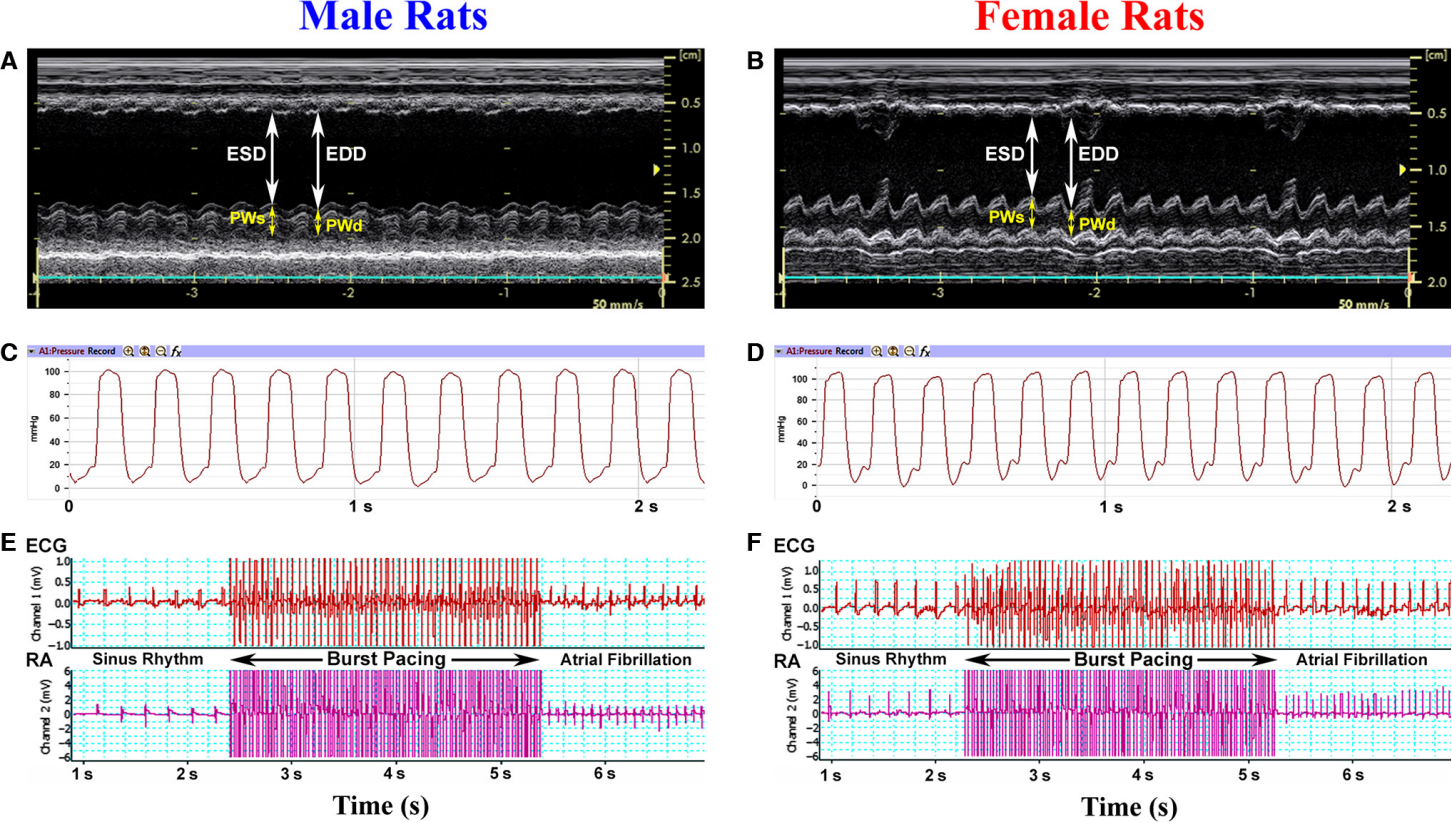
Representative recordings of M‐mode echocardiogram (A, B), LV pressure (C, D), surface electrocardiogram (ECG), and right atrial (RA) electrogram (E, F) from male and female rats 8 weeks after MI. In (E) and (F), the ECG and RA electrogram traces show the example of atrial fibrillation induction after burst pacing (for details see Material and Methods).

The measurements completed at the end of the experimental period demonstrated that post‐MI females had significantly greater LV systolic performance and function than male rats, considering the higher levels in stroke volume, stroke work, cardiac output, EF, and FS in the former (Fig. 4A, B and Table 1). At the same time, such sex‐related difference in LV systolic properties was not associated with modified ventricular contractility in females, since the maximum rate of pressure rise (d*P*/d*t* max) as well as the peak of systolic pressure were analogous between male and female post‐MI rats (Fig. 4C, D and Table 1). Nevertheless, female rats revealed a significantly lower degree of LV end‐systolic wall stress compared to males.

**Table 1:** Systolic properties of the left ventricle in male and female rats 8 weeks after MI

	M‐MI	F‐MI
*n*	6	7
Heart Rate, beats/min	318.7 ± 8.1	324.6 ± 5.2
Mean Arterial Pressure, mmHg	87.5 ± 3.4	93.6 ± 3.3
End‐Diastolic Volume, mL	0.57 ± 0.08	0.46 ± 0.02*
End‐Diastolic Volume/BW, mL/kg	0.95 ± 0.13	1.11 ± 0.09
End‐Systolic Volume, mL	0.43 ± 0.10	0.28 ± 0.03*
End‐Systolic Volume/BW, mL/kg	0.72 ± 0.19	0.68 ± 0.08
Stroke Volume, mL	0.14 ± 0.02	0.18 ± 0.01*
Stroke Work, mmHg • mL	12.1 ± 1.9	17.1 ± 0.9*
Stroke Work Index, mmHg • mL/kg	20.2 ± 3.3	40.4 ± 2.1***
Cardiac Output, mL/min	44.6 ± 8.7	57.8 ± 3.1*
Cardiac Index, mL/min/kg	74.9 ± 14.7	139.2 ± 7.9**
Fractional Shortening, %	13.3 ± 4.1	20.6 ± 2.1*
Ejection Fraction, %	26.7 ± 7.7	39.9 ± 3.4*
LV Peak Systolic Pressure, mmHg	98.6 ± 1.7	99.4 ± 3.6
Maximum d*P*/d*t*, mmHg/sec	4693.3 ± 402.4	5008.9 ± 258.5
End‐Systolic Wall Stress, kdynes/cm^2^	130.2 ± 8.5	110.6 ± 5.5*

Values are means ± SEM; *n*, number of rats. BW, body weight. *P <* *0.05, **P <* *0.01 and ***P <* *0.001 versus M‐MI.

On the other hand, both groups of rats had relatively comparable LV diastolic properties (Table 2), including a level of end‐diastolic wall stress, though the residual myocardium of male LV chamber revealed a trend to slower relaxation, suggesting some reduction in diastolic compliance.

**Table 2:** Diastolic properties of the left ventricle in male and female rats 8 weeks after MI

	M‐MI	F‐MI
*n*	6	7
End‐Diastolic Pressure, mmHg	26.4 ± 6.0	24.9 ± 2.3
End‐Diastolic Wall Stress, kdynes/cm^2^	24.7 ± 6.1	24.5 ± 3.9
Minimum d*P*/d*t*, mmHg/sec	−3193.4 ± 464.5	−4023.2 ± 304.9
Tau (Weiss), msec	17.7 ± 3.2	14.2 ± 1.6
Tau (Glantz), msec	26.3 ± 8.7	16.2 ± 1.3*

Values are means ± SEM; *n*, number of rats. Note that the time constant for isovolumic relaxation (Tau) was determined by both Tau‐Weiss method (regression of the natural logarithm of LV pressure versus time) and Tau‐Glantz method (regression of −d*P*/d*t* versus LV pressure). *P < 0.05 versus M‐MI.

### Cardiac passive electrical properties

In order to determine whether sex‐specific differences in cardiac function and performance could to some extent be caused by the distinct alterations in passive electrical properties of the remodeled heart, the atrial fibrillation (AF) inducibility tests were performed in two post‐MI groups (Fig. 4E, F). However, the rats of both sexes had relatively comparable rate of AF inducibility (~33% in males vs. ~40% in females) as well as the mean duration of induced AF episode (~3.6 sec. in males vs. ~3.2 sec in females). Furthermore, the atrioventricular conduction time was also identical in post‐MI male and female rats (59.0 ± 4.1 msec and 56.4 ± 3.3 msec, respectively). All together, these data indicate that cardiac passive electrical properties remained similar between two sexes.

### Compensatory LV hypertrophy

The postmortem examination revealed that the rats of two sex groups had relatively similar mean size of MI relative to both LV circumference and the length of LV free wall (Table 3). Furthermore, although female rats had significantly smaller ventricular weight compared to males, the degree of compensatory ventricular enlargement in response to a similar‐sized MI was comparable between sexes, as was determined on the basis of almost identical values of LV weights normalized to either body weights or tibia length. Moreover, considering the analogous values for the LV weight‐to‐total ventricular weight ratio, the interventricular scale of growth was also similar between post‐MI male and female rats (Table 3).

**Table 3:** Size of MI and the degree of LV hypertrophy in male and female rats 8 weeks after MI

	M‐MI	F‐MI
*n*	6	7
Infarct Size, % of LV	41.3 ± 3.0 (34.4–46.8)	38.1 ± 1.8 (35.4–42.9)
Infarct Size, % of LV free wall	62.5 ± 3.8 (52.6–72.4)	55.5 ± 2.3 (54.0–63.2)
VW, mg	1374.6 ± 48.9	1052.4 ± 65.8***
LVW, mg	1031.5 ± 39.3	824.9 ± 51.1**
BW, g	530.5 ± 30.3	424.3 ± 36.6**
TL, mm	48.2 ± 0.3	41.2 ± 0.6***
LVW/BW, mg/g	1.98 ± 0.14	1.97 ± 0.08
LVW/TL, mg/mm	21.2 ± 1.0	20.1 ± 1.4
LVW/VW, mg/mg	0.75 ± 0.03	0.79 ± 0.02

Values are means ± SEM; *n*, number of rats; VW, ventricular weight; LVW, left ventricular weight; BW, body weight; TL, tibia length. To assess LV size independently of the effect of significantly different body size between two sex groups, LV weight was normalized to body weight and tibia length. **P <* *0.01 and ***P <* *0.001 versus M‐MI.

### LV chamber structural parameters and remodeling indices

**Figure 5:**
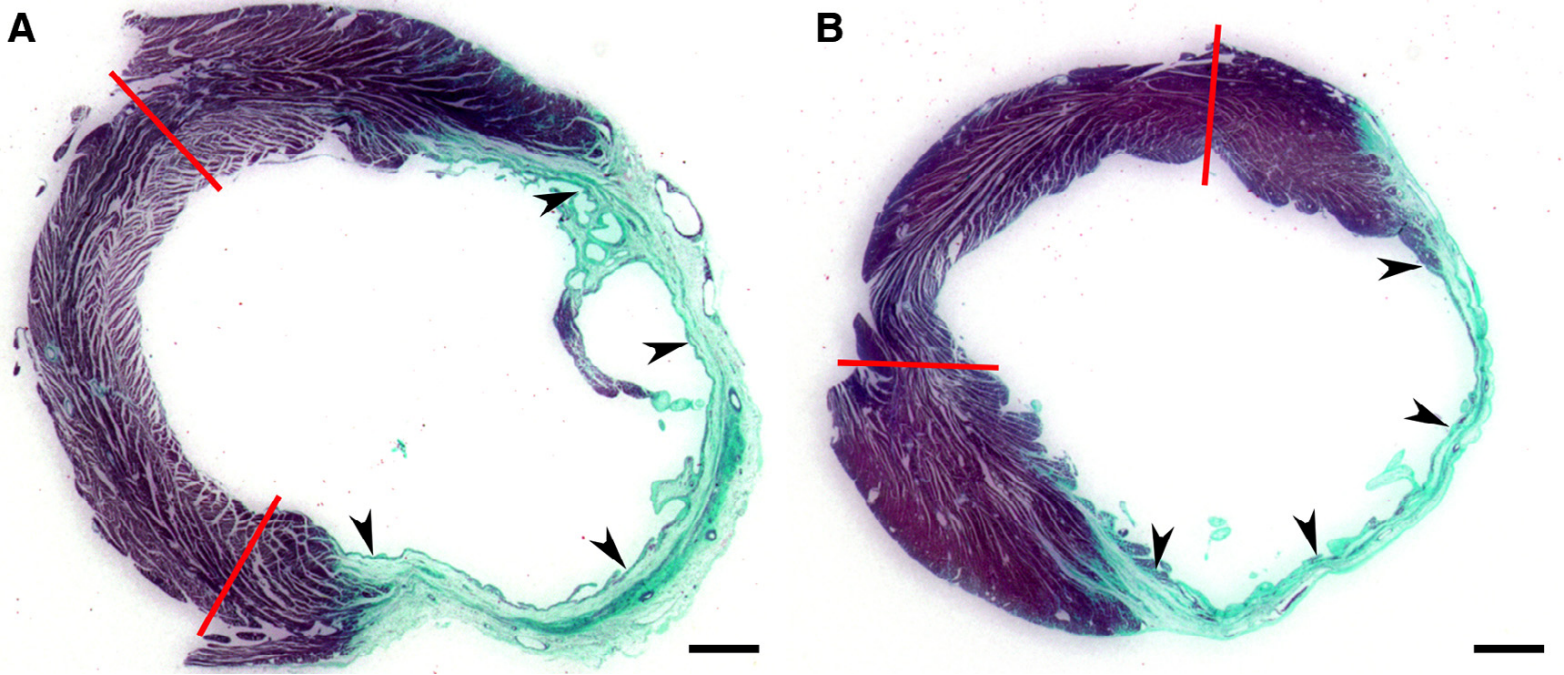
Representative images of Masson's trichrome‐stained transverse sections of the LV chamber from male (A) and female (B) rats 8 weeks after MI. Since right ventricular free wall has been removed during heart processing, the red lines mark the borders between the interventricular septum and LV free wall. Note that in both male and female hearts a large area of LV free wall is occupied by the fibrous transmural scar (arrowheads). Scale bars are 1.2 mm.

The morphological evaluation of the left ventricles revealed that although the transverse size of LV chamber as well as LV cavity was significantly smaller in females than in male rats, the average wall thickness in different LV regions, such as septum, noninfarcted free wall, and transmural scar, were relatively similar between two sex groups (Fig. 5). Most important, the conventional indices of LV chamber remodeling were generally comparable between rats of opposite sexes (Table 4), indicating the analogous scale of LV cavity expansion relative to regional changes in ventricular wall thickness.

**Table 4:** Structural LV parameters and chamber remodeling indices in male and female rats 8 weeks after MI

	M‐MI	F‐MI
*n*	6	7
LV chamber diameter, mm	11.2 ± 0.3	10.4 ± 0.4*
LV chamber CSA, mm^2^	100.2 ± 5.5	85.6 ± 6.1*
LV cavity diameter, mm	7.5 ± 0.5	6.1 ± 0.5*
LV cavity CSA, mm^2^	45.4 ± 5.5	30.1 ± 5.0*
LV free wall thickness, mm	2.8 ± 0.2	2.9 ± 0.2
Septum thickness, mm	2.4 ± 0.2	2.4 ± 0.2
Scar thickness, mm	0.9 ± 0.1	0.8 ± 0.1
Scar thinning index	0.41 ± 0.03	0.32 ± 0.05
LV cavity diameter‐to‐Septum ratio	3.27 ± 0.52	3.01 ± 0.59
LV cavity expansion index	1.01 ± 0.06	1.23 ± 0.12

Values are means ± SEM; *n*, number of rats; CSA, cross‐sectional area. Scar thinning ratio = scar thickness/septal wall thickness; LV cavity expansion index = (LV cavity area/LV chamber area) × (septal wall thickness/scar thickness). Because for histological examination, hearts were collected in the dilated state, the values of the structural parameters listed in this table can be considered as obtained in the state near the end of diastole. Note that LV free wall thickness was estimated as a mean of the averages determined in noninfarcted parts of the LV anterior and posterior walls. *P <* *0.05 versus M‐MI.

### Myocardial fibrosis and mast cell density

Considering the fact that changes in the content of fibrillar collagen within remaining LV myocardium could markedly affect ventricular wall compliance, the volume fraction of interstitial collagen was compared between post‐MI male and female rats. However, the rats of both sexes had relatively comparable levels of fibrillar collagen within the myocardial interstitium of two examined regions: the noninfarcted free wall and the septum (Table 5). Moreover, the interstitial density of mast cells, one of the key mediators of myocardial fibrosis, remained similar between sexes in both LV regions.

**Table 5:** Collagen content and numerical density of mast cells in noninfarcted LV myocardium of male and female rats 8 weeks after MI

	*n*	Interstitial collagen volume fraction (%)	Mast cell density (cells/mm^2^)
LV Free Wall			
M‐MI	6	14.3 ± 1.2	1.65 ± 0.41
F‐MI	7	12.9 ± 0.8	1.74 ± 0.36
Septum			
M‐MI	6	12.6 ± 1.1	1.48 ± 0.22
F‐MI	7	11.6 ± 0.7	1.68 ± 0.24

Values are the means ± SEM; *n*, number of rats.

### LV cardiac myocytes and myocardial capillaries

**Figure 6:**
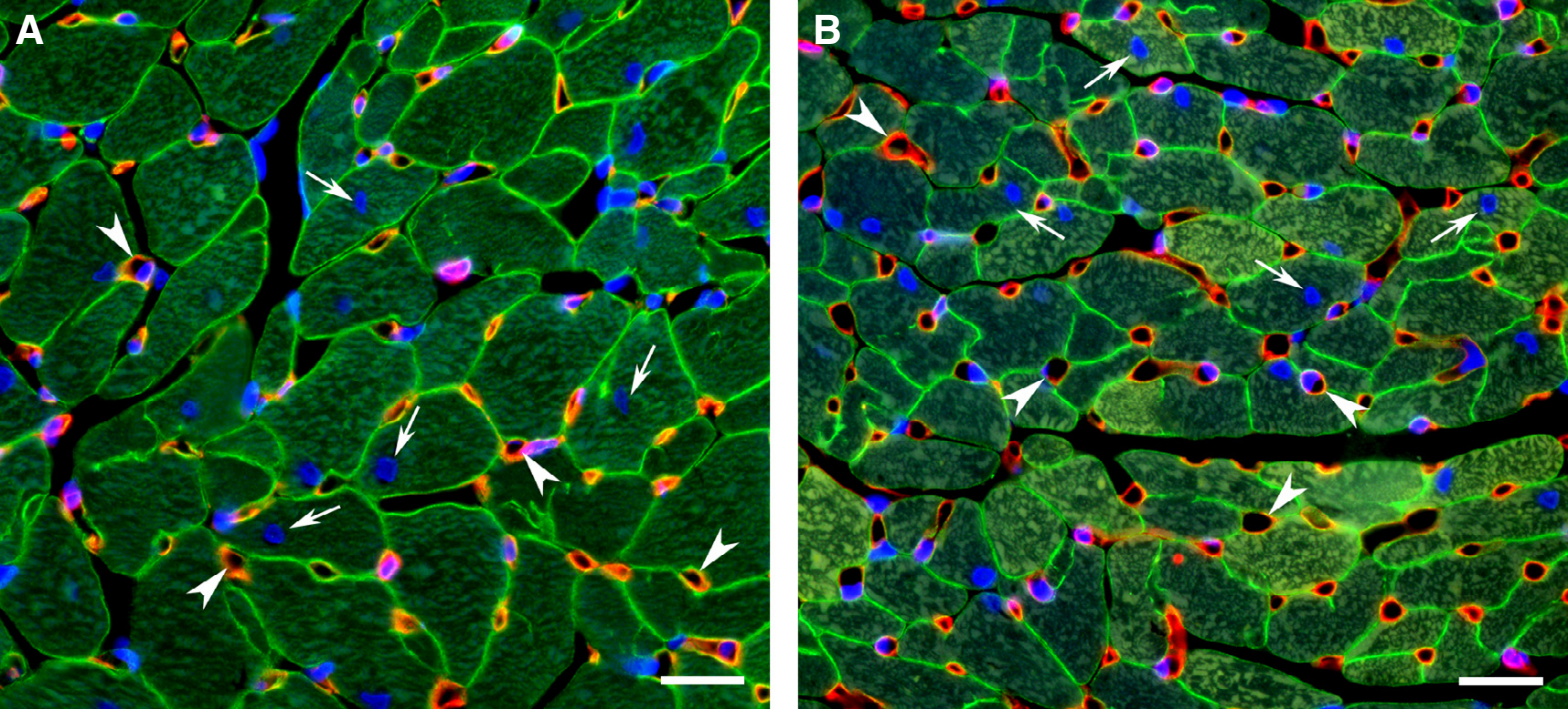
Representative images from the noninfarcted epimyocardial region of the LV free wall myocardium from male (A) and female (B) rats 8 weeks after MI. The transverse sections of the LV chamber were immunostained against laminin (green color) to outline the cross‐sectioned profiles of cardiac myocytes and co‐labeled with *Griffonia Simplicifolia* isolectin IB4 (red color) to visualize the endothelial lining of the blood capillaries. DAPI stain (blue) was used to counterstain the nuclei. Note that the quantitative evaluation of structural parameters describing the capillary network and the size of cardiac myocytes was done only in the areas in which the capillaries had the transverse profiles (arrowheads) while the cardiac myocytes showed the round‐shaped, centrally located nuclei (arrows). Scale bars are 20 *μ*m.

**Figure 7:**
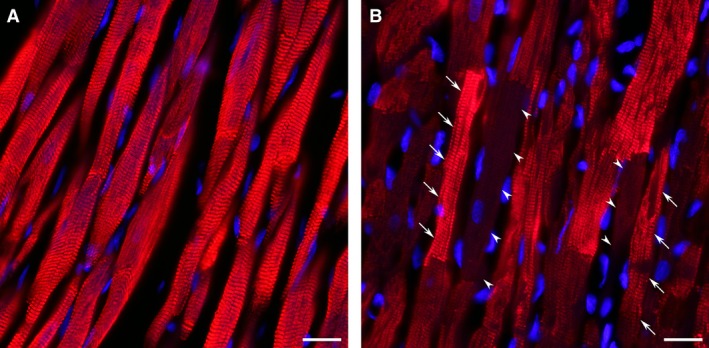
Representative images from the remaining noninfarcted myocardium of the LV free wall immunostained against cardiac MHC‐*β* isoform (red color) from male (A) and female (B) rats 8 weeks after MI. DAPI stain (blue) was used to counterstain the nuclei. Note that in contrast to male myocardium, in which the cardiac myocytes reveal almost identical level of MHC‐*β* isoform expression (A), the cardiac myocytes in female heart demonstrate a high level of heterogeneity (B). In female myocardium, the arrowheads indicate the cardiac myocytes with a low level of MHC‐*β* isoform expression, whereas the arrows point out the cells with the high level of protein expression. Scale bars are 20 *μ*m.

Since the size and density of cardiac myocytes within the ventricular wall as well as the extent of supporting capillary network are the primary determinants of cardiac contractile performance, any noticeable differences in these parameters could have a marked effect on LV function. Although, in the interventricular septum, both groups of post‐MI rats demonstrated relatively similar size of cardiac myocytes and the extent of the capillary bed, in the remaining myocardium of the LV free wall, female rats had significantly smaller, than males, the mean diameter and cross‐sectional area of cardiac myocytes, specifically, within the epimyocardial region (Fig. 6 and Table 6). The smaller enlargement of cardiac myocyte in the female free wall myocardium was associated with the lack of noticeable reactive coronary angiogenesis, as was confirmed by a significantly smaller, than in males, capillary‐to‐myocyte ratio (Table 6). Most important, such sex‐related difference in the average width of free wall cardiac myocytes between post‐MI male and female rats resulted in a markedly higher regional cardiac myocyte density in female myocardium. Taking into account that rats of both sexes demonstrated similar thickness of the noninfarcted free wall (Table 4), it became evident that female myocardium, in comparison to males, had a significantly higher cardiac myocyte density across the ventricular wall. Furthermore, while in female LV myocardium, only individual cardiac myocytes showed an increased level of cardiac MHC‐*β* isoform expression, in male rats, most of surviving cardiac myocytes revealed the homogeneously high scale of cardiac MHC‐*β* isoform production (Fig. 7), indicating a more advanced degree of the adverse molecular changes in hypertrophied myocardium of males compared to female rats.

**Table 6:** Cardiac myocyte size, capillary and cardiac myocyte numerical densities, and capillary‐to‐myocyte ratio in the left ventricle of male and female rats 8 weeks after MI

	*n*	Myocyte Diameter, *μ*m	Myocyte CSA, *μ*m^2^	CD, number/mm^2^	MD, number/mm^2^	C/M
Free Wall Epimyocardium						
M‐MI	6	16.3 ± 0.4	479.0 ± 20.6	3029.9 ± 95.6	1785.7 ± 55.9	1.75 ± 0.09
F‐MI	7	14.0 ± 0.3***	373.5 ± 19.1**	3369.5 ± 173.1	2383.8 ± 242.6*	1.48 ± 0.07*
Septal Endomyocardium						
M‐MI	6	12.9 ± 0.4	348.4 ± 24.5	3726.3 ± 240.7	2485.8 ± 187.8	1.53 ± 0.06
F‐MI	7	12.2 ± 0.4	309.5 ± 16.8	3944.9 ± 272.8	2842.9 ± 156.1	1.40 ± 0.06

Values are means ± SEM. *n*, number of rats; CSA, cross‐sectional area; CD, capillary density; MD, myocyte density; C/M, capillary‐to‐myocyte ratio. Note that in septum all parameters were analyzed in endomyocardium only on the side of the left ventricle. *P <* *0.05, **P <* *0.01 and ***P <* *0.001 versus M‐MI.

### Myocardial apoptosis

**Figure 8:**
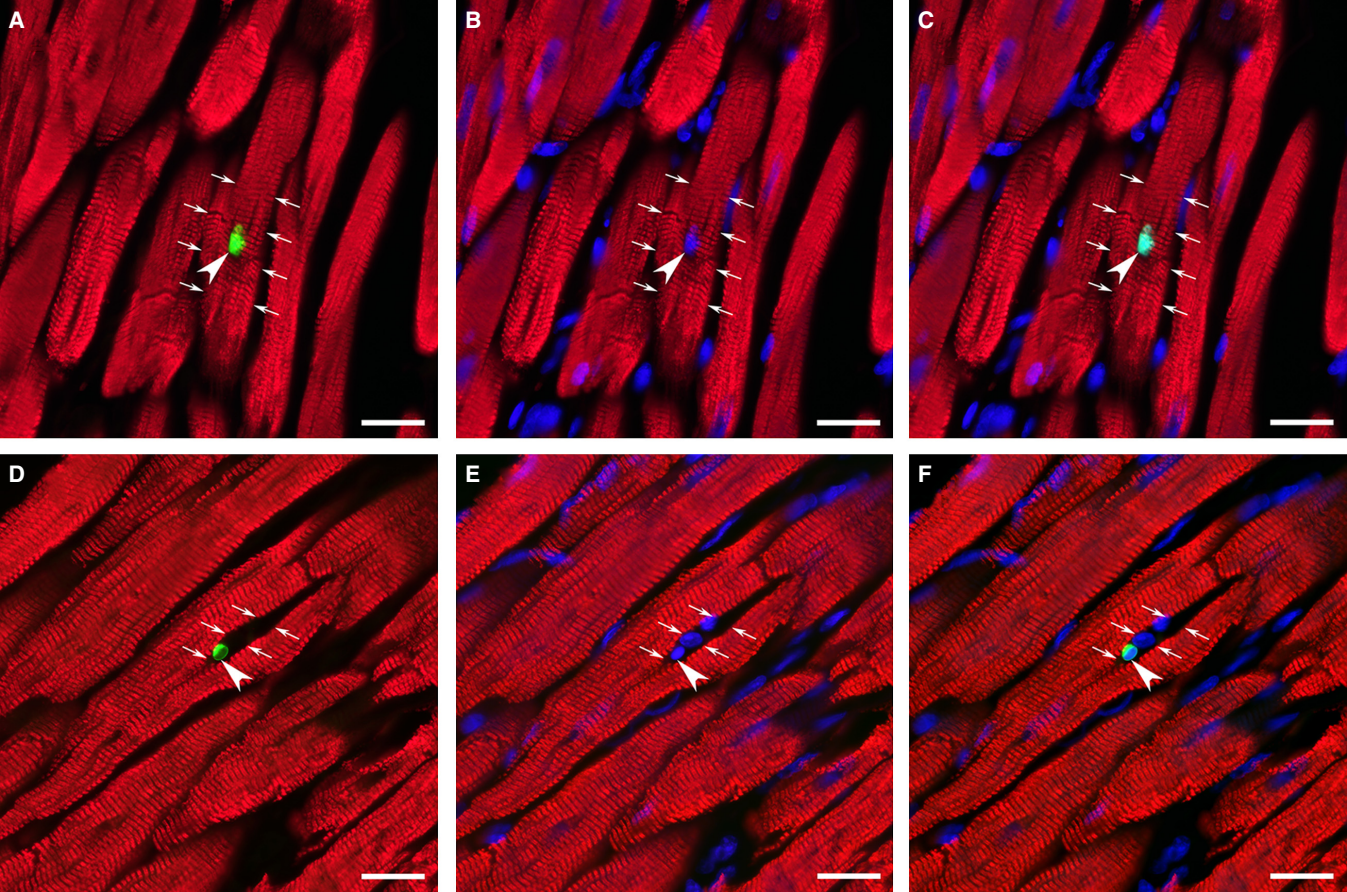
Representative images of the TUNEL‐positive nuclei (green color) in a cardiac myocyte (A, B, C) immunostained against cardiac MHC‐*β* isoform (red color) and a noncardiac myocyte cell (D, E, F) in noninfarcted LV myocardium of post‐MI heart. DAPI stain (blue) was used to counterstain the nuclei. In (A), (B) and (C), the arrows outline the same apoptotic cardiac myocyte, while the arrowheads points to a TUNEL‐positive nucleus. In (D), (E) and (F), the arrows outline interstitial space between the adjacent cardiac myocytes, while the arrowheads indicate a TUNEL‐positive nucleus in the same noncardiac myocyte cell. Note that the pool of myocardial noncardiac myocyte cells (immunonegative for a cardiac MHC‐*β* isoform antibody) might include various interstitial, inflammatory, and microvascular cell types. Scale bars are 20 *μ*m.

**Figure 9:**
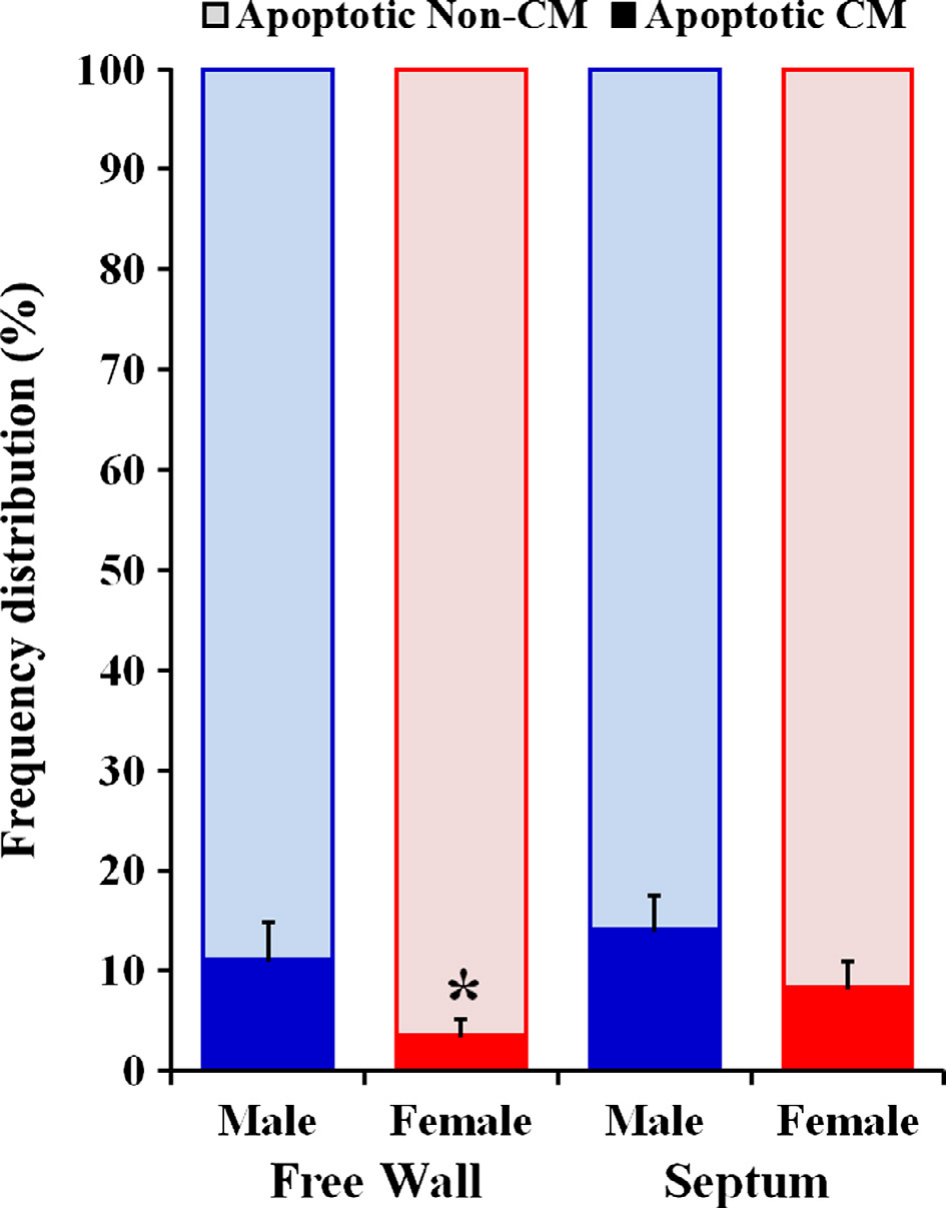
Frequency distribution of apoptotic (TUNEL‐positive) cardiac myocytes (CM) and noncardiac myocyte (non‐CM) cells in remaining noninfarcted myocardium of the LV free wall and interventricular septum of post‐MI male and female rats. Data are means ± SEM;* n* = 6 male rats/group; *n* = 7 female rats/group. *P < 0.05 F‐MI versus M‐MI rats.

In view of the fact that a marked difference in cardiac myocyte density between post‐MI male and female rats could be a consequence of the various extents in cardiac myocyte loss, particularly via apoptotic death, the density of TUNEL positive cells was determined in remaining LV myocardium of rats from both sex groups. The TUNEL assay demonstrated that in both post‐MI groups, there were two distinct pools of TUNEL positive cells: (1) apoptotic cardiac myocytes (Fig. 8A–C); and (2) apoptotic noncardiac myocyte (non‐CM) cells (Fig. 8D–F). The analysis of frequency distribution between these two apoptotic cell types revealed that the apoptotic non‐CM cells were always predominant in both sexes (Fig. 9). At the same time, compared to male rats, females had a significantly smaller pool of apoptotic cardiac myocytes, especially, in the residual myocardium of the LV free wall, as indicated a clear downshift in frequency distribution between two groups of cells (Fig. 9). Most important, the remaining LV myocardium of post‐MI female rats demonstrated a significantly lower, than in males, spatial tissue density of apoptotic cardiac myocytes (Table 7).

**Table 7:** Numerical density of apoptotic (TUNEL‐positive) cardiac myocytes (CMs) and noncardiac myocyte (Non‐CM) cells in noninfarcted LV myocardium of male and female rats 8 weeks after MI

	*n*	CMs (cells/mm^2^)	Non‐CM cells (cells/mm^2^)
LV Free Wall			
M‐MI	6	0.91 ± 0.30	7.39 ± 1.43
F‐MI	7	0.18 ± 0.08**	5.72 ± 0.67
Septum			
M‐MI	6	1.15 ± 0.17	8.55 ± 1.81
F‐MI	7	0.33 ± 0.09***	4.60 ± 0.68*

Values are the means ± SEM; *n*, number of rats. *P <* *0.05, **P <* *0.01 and ***P <* *0.001 versus M‐MI.

## Discussion

The results of this study provide several important findings concerning the effect of sex on the pattern of dynamic changes in cardiac function associated with MI‐induced alterations in LV chamber geometry and intrinsic myocardial properties in middle‐aged rats. First, we found that although the general pattern of cardiac functional decline caused by a large, similar‐sized MI was analogous in two sex groups, female rats have shown significantly greater, than in males, LV systolic function and cardiac performance at rest and for the duration of an entire post‐MI period. To a great extent, the poorer pumping capacity of the left ventricle in post‐MI males was a result of the lower, than in female rats, diastolic compliance rather than more severe contractile impairment. Second, in both sex groups, a large, transmural MI triggered analogous eccentric remodeling of the LV camber. However, while in female rats, the dynamic of changes in LV geometry remained relatively similar for the duration of a post‐MI period, in male rats, the left ventricle has acquired a structural pattern consistent with eccentric hypertrophy somewhere between 6 and 8 weeks after MI. A switch in LV geometry, seen in male rats during this period, has coincided with more enhanced, compared to females, growth of the residual myocardium. Third, we determined that, by the end of eighth post‐MI week, two sexes were comparable by the scale of structural LV remodeling, the extent of myocardial fibrosis, the levels of ventricular contractility, and the state of passive electrical properties, suggesting that these parameters could not be accounted for better systolic function in the female left ventricle. At the same time, in males, the left ventricle demonstrated an evident trend to a prolonged isometric relaxation that was coincided with the uniformly high production of a cardiac MHC‐*β* isoform in remaining male cardiac myocytes. Finally, the surviving female myocardium of LV free wall revealed a higher, than in males, numerical density of cardiac myocytes, specifically, in epimyocardial region. This phenomenon was associated with a markedly lower spatial tissue density of apoptotic cardiac myocytes in female rats. Altogether, our current findings determined that during progressive MI‐induced LV remodeling the female middle‐aged rats have been able to maintain markedly better systolic function and cardiac performance compared to male counterparts primarily due to the fact that the remaining female myocardium had functionally favorable intrinsic tissue properties.

### Does MI‐induced remodeling alter LV geometry and function in a sex‐dependent manner?

A number of clinical and experimental animal (predominantly rodent) studies has previously demonstrated that a large transmural MI causes significant structural alterations in LV chamber, leading to marked cardiac dysfunction and, eventually, to systolic HF (Pfeffer and Braunwald 1990; Weir et al. 2006). In response to contractile dysfunction and increased hemodynamic load, the post‐MI heart in both humans (Gaasch and Zile 2011) and rodents, specifically rats (Olivetti et al. 1991; Pfeffer et al. 1991) and mice (Patten et al. 1998; Yang et al. 2002), undergoes the analogous transformations, comprising LV chamber dilatation and the compensatory growth of residual myocardial tissue. Because the scale of post‐MI ventricular hypertrophy, in most cases, remains inadequate to match the extent of LV cavity dilatation (Pfeffer et al. 1991; Litwin et al. 1994), the LV chamber has typically acquired the geometry consistent with eccentric remodeling (Gaasch and Zile 2011). Although such alteration has worsened systolic dysfunction, it helps attenuate the decline in cardiac performance by preserving stroke volume and, thereby, cardiac output (Pfeffer et al. 1979, 1984). Our current findings obtained on post‐MI middle‐aged rats of both sexes were highly consistent with these observations. However, while we determined that the dynamic changes in overall pattern of LV remodeling and the decline in global and regional systolic function have been analogous between male and female rats during the first 6 weeks following a large, similar‐sized MI, noticeable sex‐related differences in LV geometry and cardiac performance were found between two sex groups at the end of eighth post‐MI week.

Although it becomes widely accepted that biological sex has a profound impact on cardiac remodeling in both humans and animals, including that caused by ischemia or an MI (Ostadal et al. 2009; Piro et al. 2010; Dunlay and Roger 2012), the detailed mechanisms of these sex‐related differences have remained mostly unknown. Even among studies, which used a standard murine model of permanent coronary artery ligation to induce an MI, the extent and the precise nature of sex‐related influence continue to be highly controversial. For instance, among experimental studies, employing rats or mice of both sexes as the subjects for a large transmural MI, there were some which could not find any impact of sex on structural and functional LV remodeling (Bridgman et al. 2005; Chen et al. 2011; Antonio et al. 2015), whereas others have reported the existence of sex‐related differences only in one type of LV parameters, such as the internal dimensions (Sofia et al. 2014), chamber geometry (Litwin et al. 1999), or systolic function (Shioura et al. 2008). Surprisingly, only two previous studies by Cavasin et al. (Cavasin et al. 2004) and Wang et al. (Wang et al. 2007) have reported that female mice compared to males had less severe changes in both LV structural remodeling and systolic dysfunction following a large MI.

In this regard, it is important to emphasize that, in our current study, we did observe two fundamental sex‐related differences associated with structural and functional alterations in the left ventricle of the middle‐aged rats following a large, similar‐sized MI. First, similar to findings by Cavasin et al. (Cavasin et al. 2004) on young‐adult mice of both sexes, we determined that middle‐aged female rats compared to their age‐matched male counterparts had significantly better global systolic function for the entire duration of the study. Second, in agreement with data reported earlier by Litwin et al. (1999), we revealed that male rats, in contrast to females, were capable of additional LV hypertrophy in time after MI. However, in contrast to this previous report completed on young‐adult rats, in our study, the advanced increase in LV mass, including the thickening of the posterior wall myocardium, had occurred later in remodeling process, somewhere between six and eight post‐MI weeks. As a result, we found that by the end of eighth post‐MI week, the male LV chamber has acquired the geometry more consistent with eccentric hypertrophy, whereas geometry of female LV chamber remained relatively the same between weeks two and eight after MI. To some extent, the later onset of such advanced post‐MI hypertrophy in male myocardium of the middle‐aged rats might be due to the fact that our animals were older, than those used previously by other investigators, and that their myocardial growth reserve could be modified by aging (Cheng et al. 1996b).

Most interesting, the noticeable improvement of global LV geometry in post‐MI male middle‐aged rats did not translate into the enhancement of their cardiac pumping capacity. Considering our current data, showing significantly lower, than in females, diastolic compliance in the male left ventricle, as well as the analogous findings reported earlier by Litwin et al. (1999), it is feasible to suggest that poorer cardiac function in post‐MI male middle‐aged rats might be a result of higher, than in females, LV wall rigidity. Recognizing that the increased stiffness of LV wall in combination with reduced ventricular relaxation could markedly limit the capacity of the male ventricle to expand EDV and, thereby, the stroke volume, we believe that such difference in comparison to females could be the key factor that impaired capability of male heart to maintain EF and CO, especially, under the circumstance when the ventricular contractility has been markedly diminished to the similar extent in two sexes. The latter assumption is partially supported by the previously reported findings, which demonstrated that the heart of young‐adult male rats, contrary to age‐matched females, has adapted to physical training, that is, to elevated hemodynamic loading, principally by an increase in contractile performance of the left ventricle (Schaible et al. 1981). Although it must be recognized that to some extent such sex‐specific adaptation of cardiac function to increased loading condition could be a result of the difference in size between larger male and smaller female hearts (Schaible et al. 1981; Schaible and Scheuer 1984), it has been established that even when cardiac function was compared between age‐matched male and female rats with the similar‐sized hearts, it still appeared that male rats had better cardiac function compared to females predominantly because of greater contractile performance (Schaible et al. 1981). In this regard, it is also important to emphasize that during exercise the healthy middle‐aged men increased stroke volume primarily by increasing ventricular contractility, whereas age‐matched women used mainly an increase in the end‐diastolic volume in order to achieve the same results (Higginbotham et al. 1984). Therefore, it appears that sex‐related difference in the ability of middle‐aged rats to regulate the LV stroke volume and, hence, cardiac performance during MI‐induced remodeling in two specific ways could reflect a basic distinction between male and female cardiac response to hemodynamic overload.

Our current experimental findings on middle‐aged rats were very similar to clinical studies in humans, which demonstrated that women with systolic HF had a lesser degree of maladaptive LV hypertrophy (Crabbe et al. 2003) and, most importantly, higher LV ejection fraction (O'Meara et al. 2007) compared to male counterparts. Such sex‐related advantage of females over the male patients with post‐MI or ischemic systolic HF has been often linked to more favorable remodeling process in the female heart that facilitated the delay of cardiac decompensation in women (Piro et al. 2010; Dunlay and Roger 2012). In concert with these clinical observations, our current results as well as experimental findings reported previously by others on mice (Cavasin et al. 2004; Shioura et al. 2008) have indicated that cardiac function in post‐MI male animals continued to deteriorate compared to post‐MI females even during the time when the male LV myocardium has undergone the additional growth. Hence, the absence of noticeable alleviation of systolic impairment in the hypertrophied left ventricle of post‐MI male middle‐aged rats, has suggested that sex‐specific changes in internal tissue properties of the surviving myocardium rather than alterations in LV geometry could primarily underlie sex‐related differences in LV function and cardiac performance in male and female rats after MI.

### Can sex influence myocardial tissue properties during MI‐induced LV remodeling?

In the last several decades, MI‐induced myocardial tissue remodeling remained one of the intensely studied area, particularly, because of its importance in development of novel therapeutic modalities in treatment of LV systolic dysfunction (Pfeffer and Braunwald 1990; Michel et al. 1995; Abbate et al. 2002; Dedkov et al. 2005, 2007, 2015). According to numerous studies, the surviving LV myocardium of post‐MI hearts in both humans and animals undergoes relatively similar tissue alterations, including interstitial/perivascular fibrosis (Beltrami et al. 1994; Cavasin et al. 2004; Bridgman et al. 2005; Dedkov et al. 2007), loss of cardiac myocytes via apoptosis (Sam et al. 2000; Palojoki et al. 2001; Baldi et al. 2002), compensatory enlargement of the remaining cardiac myocytes (Olivetti et al. 1991; Beltrami et al. 1994; Cavasin et al. 2004; Bridgman et al. 2005; Dedkov et al. 2006, 2007), reactive angiogenesis, and/or arteriogenesis (Dedkov et al. 2006, 2014). In addition, some reports have also revealed the existence of regional differences in adaptive responses of major tissue components in the remaining LV myocardium (Capasso et al. 1992; Dedkov et al. 2006, 2014), which for the most part were related to an uneven distribution of the increased hemodynamic stress on the remodeled ventricular wall in the post‐MI heart (Capasso et al. 1992; Anversa et al. 1993; Rohde et al. 1999).

Most recently, in view of the growing number of clinical and experimental animal studies reporting noticeable sex‐related differences in post‐MI myocardial remodeling (Piro et al. 2010), it has been proposed that sex‐specific changes in intrinsic myocardial properties could be an essential factor responsible for worse clinical outcome among male, as opposed to female, patients with ischemic or post‐MI systolic HF (Dunlay and Roger 2012). Unfortunately, because only a handful of these studies has explored this matter in sufficient depth (Guerra et al. 1999; Litwin et al. 1999; Crabbe et al. 2003; Cavasin et al. 2004; Biondi‐Zoccai et al. 2005; Bridgman et al. 2005; Chen et al. 2011; Dedkov et al. 2014), the true nature (onset, duration, intensity or scale) of such sex‐related changes has often remained controversial.

For instance, the earlier findings by Cavasin et al. (Cavasin et al. 2004) and Bridgman et al. (Bridgman et al. 2005) demonstrated that young‐adult (~3‐month‐old) male and female mice have undergone a very similar scale of interstitial collagen accumulation in residual LV myocardium during 12 and 6 weeks following a large MI, respectively. On the other hand, our previous study on middle‐aged (~12‐month‐old) rats revealed that female LV myocardium had a markedly smaller amount of interstitial collagen, compared to males, both in the absence of coronary artery ligation and 4 weeks after a similar‐sized MI. However, in present study, we found that by the end of eight post‐MI week, the same middle‐aged rats have demonstrated almost identical extent of myocardial fibrosis between two sexes. Taking into consideration the fact that aged male rats always had more advanced myocardial fibrosis compared to age‐matched females (Forman et al. 1997; Dedkov et al. 2014), our current finding suggests that during progression of MI‐induced cardiac remodeling, female middle‐aged rats might undergo more intense, than males, collagen deposition in surviving LV myocardium. Nevertheless, despite probable sex‐related differences in the rate of interstitial collagen production in the remaining LV myocardium, we believe that the changes in the volume fraction of fibrillar collagen could not account for markedly better functional performance detected in middle‐aged female rats during 8 post‐MI weeks.

At the same time, current results concur with our previous findings, demonstrating that post‐MI middle‐aged female rats had higher density of cardiac myocytes in the epimyocardium of the noninfarcted LV free wall compared to male rats (Dedkov et al. 2014). Most important, a noticeable decrease in cardiac myocyte density within epimyocardial region of post‐MI males was primarily caused by a significant transverse enlargement of the remaining cells. According to a well‐established fact that in post‐MI rats, a spatial pattern of cardiac myocytes hypertrophy within LV myocardium followed a clear transventricular wall gradient, with larger cells in endomyocardium and smaller in epimyocardium, due to gradual distribution of ventricular wall stress (Olivetti et al. 1991; Capasso et al. 1992; Anversa et al. 1993), it becomes evident that in our post‐MI middle‐aged male rats, epimyocardial cardiac myocytes could grow bigger principally as a result of higher, than in females, LV end‐systolic wall stress. Unfortunately, this assumption is difficult to corroborate since almost all previously published experimental studies, which examined sex‐related changes in the remaining cardiac myocytes of post‐MI hearts (Litwin et al. 1999; Cavasin et al. 2004; Bridgman et al. 2005; Chen et al. 2011), except our own (Dedkov et al. 2014), did not specify the exact topographic LV regions in which morphometric analyses were completed. Consequently, although several earlier reports have similarly indicated that the male cardiac myocytes in post‐MI mice (Cavasin et al. 2004), rats (Litwin et al. 1999) and humans (Crabbe et al. 2003) were larger than in females, there is a high degree of uncertainty with regard to the results of some other studies, which have been unable to detect such sex‐related differences in post‐MI myocyte growth (Bridgman et al. 2005; Chen et al. 2011).

Despite such inconsistencies among the above‐mentioned studies, our previous (Dedkov et al. 2014) as well as current observations have repeatedly demonstrated that in post‐MI middle‐aged rats, the surviving female myocardium contained a significantly greater, than in males, number of cardiac myocytes across the same width noninfarcted LV free wall. We presume that this fact could explain a favorable advantage of the post‐MI female heart in supporting better LV function and cardiac performance, compared to males, under the condition of analogous, between sexes, hemodynamic overload. We may consider two potential mechanisms which can be accountable for sex‐related differences in cardiac myocyte density. At first, taking into account that, in our study, post‐MI middle‐aged female rats, in contrast to males, had markedly lower number of apoptotic cardiac myocytes within surviving LV myocardium, it is feasible to suggest that the female myocardium has experienced a lesser, than in males, degree of structural alterations in cardiac myocyte syncytium. Since, it has been previously suggested that elevated mechanical stretch on the residual myocardium could induce programmed death in some cells in order to facilitate the rearrangement of cardiac myocytes in remodeling LV chamber (Cheng et al. 1995, 1996a; Anversa et al. 1998), we hypothesize that higher LV end‐systolic wall stress detected in our post‐MI middle‐aged male rats may to some extent explain a significant increase in apoptotic rate among male cardiac myocytes that was necessary for further modification of LV chamber geometry. This assumption can be strengthened by the fact that elevated regional wall stress in the remaining myocardium of the post‐MI heart in rats are often correlated with higher extracellular matrix degradation activity, responsible for progressive LV remodeling (Rohde et al. 1999).

On the other hand, considering the fact that LV cardiac myocytes of adult female mice revealed a higher resistance to oxidative damage compared to male myocytes (Wang et al. 2010), it seems reasonable to hypothesize that, in contrast to males, LV cardiac myocytes in post‐MI middle‐aged female rats had better survival under condition of chronically elevated oxidative stress due to MI‐triggered functional overload of the remaining LV myocardium (Anversa et al. 1998). In accord, the absence of a noticeable angiogenesis in epimyocardium of post‐MI middle‐aged female rats, as opposed to males, has evidently indicated that, in our study, female cardiac myocytes did not produce the proangiogenic stimuli commonly associated with increased ischemic or oxidative damage.

Furthermore, it is important to emphasize that, in this study, we thoroughly distinguished apoptotic cardiac myocytes from apoptotic non‐CM cells in order to determine the true extent of myocyte death within remaining LV myocardium. Hence, in contradiction to several earlier studies, which specified that only cardiac myocytes have undergone the apoptotic death during MI‐induced remodeling (Sam et al. 2000; Palojoki et al. 2001; Zhao et al. 2004), our current findings on middle‐aged rats have confirmed the results published previously by Park et al. (Park et al. 2009), who demonstrated that in primates and humans with systolic HF apoptosis predominate in non‐CM cells. Moreover, we also determined that a sex‐based difference in the extent of cardiac myocyte death detected in post‐MI middle‐aged rats was not a consequence of enhanced aging‐associated loss of male cardiac myocytes, as opposed to aging females, as it has been previously shown in old primates (Zhang et al. 2007) and humans (Olivetti et al. 1995; Mallat et al. 2001). According to our analysis, noninfarcted age‐matched male and female rats had relatively similar extent of programmed cell death between myocytes (0.3 ± 0.2 cells/mm^2^ vs. 0.2 ± 0.1 cells/mm^2^, respectively) as well as non‐CM cells (6.8 ± 1.3 cells/mm^2^ vs. 6.4 ± 1.4 cells/mm^2^, respectively). Therefore, we believe that markedly higher regional density of cardiac myocyte discovered in residual LV myocardium of post‐MI female middle‐aged rats, as opposed to male rats, can be one of the essential sex‐related distinctions enabling female post‐MI hearts to demonstrate better cardiac function.

In addition, since we found a highly uniformed expression of cardiac MHC‐*β* isoform in the residual LV myocardium of post‐MI male middle aged rats, compared to females, it is also likely that poorer functional performance of the male heart can in part be caused by markedly prolong ventricular relaxation. Taking into consideration the fact that in experimental animal models of progressive myocardial hypertrophy, the depression of LV function was always associated with the increased production of *β*‐(slower)‐type cardiac myosin isoform (Schwartz et al. 1981; Michel et al. 1995), it seems feasible to suggest that depressed cardiac performance in post‐MI male middle‐aged rats can be a direct consequence of advanced growth of male cardiac myocytes associated with the switch in cardiac MHC from *α*‐ to, predominantly, *β*‐isoform.

### Study limitations

Considering the design of this study, it is important to mention several limitations in interpretation of our findings. It appears that LV systolic function in middle‐aged female rats was better than in age‐matched males at the beginning of the study, that is, before onset of a large MI. Therefore, taking into account the fact that female hearts were significantly smaller than male hearts throughout an entire experimental period, it is difficult to exclude the probable effect of sex‐related difference in heart size on cardiac performance during MI‐induced LV remodeling, unless the age‐matched female and male rats with the similar‐sized hearts would be also evaluated. Another limitation of this study was the fact that although cardiac function and geometry were repeatedly evaluated at several time points during post‐MI period, the intrinsic myocardial properties were examined only once at the end of experiment. Therefore, it prohibited us to perform a parallel analysis of dynamic sex‐related changes between cardiac and myocardial parameters at different time points following MI. Moreover, since we did not assess the left atrial filling pressure or pulmonary artery wedge pressure, it remained unclear whether elevated preload, in addition to better LV compliance, was also responsible for a greater stroke volume and, hence, cardiac performance in female rats compared to males. Additionally, because our study was limited by the eighth post‐MI week, there is no compelling evidence substantiating the concept that progressive eccentric remodeling in post‐MI female rats would persistently facilitate the better cardiac performance in comparison with males. Furthermore, it is important to emphasize that in this study, we did not intend to investigate the influence of sex hormones during MI‐induced remodeling process, instead, we attempted to find the potential sex‐related differences in functional and structural responses of the post‐MI male and female hearts under the natural circumstances that might occur in middle‐aged humans.

## Conclusions

The findings in our study reveal that the post‐MI middle‐aged female rats, in contrast to age‐matched males, were able to preserve the functionally favorable tissue properties of the remaining noninfarcted LV myocardium, specifically the higher density of cardiac myocytes and the lesser accumulation of *β*‐(slow‐twitch)‐isoform of cardiac‐MHC. The attenuation of myocardial remodeling during post‐MI period has allowed the female left ventricle to retain greater, than in males, compliance and, hence, a stroke volume. We believe that such sex‐related distinctions of post‐MI middle‐aged female rats enabled them to significantly reduce the decline in LV systolic function and cardiac performance compared to their male counterparts.

## Conflict of Interest

None declared.
